# A study exploring regional level predictors of suicide rates across time in Sweden

**DOI:** 10.1192/j.eurpsy.2024.59

**Published:** 2024-08-27

**Authors:** E. T. Eliasson, V. Carli

**Affiliations:** ^1^National Centre for Suicide Research and Prevention (NASP), Karolinska Institute, Solna, Stockholm, Sweden

## Abstract

**Introduction:**

In Sweden, four lives are lost to suicide each day. Hence, identifying relevant risk factors to inform effective prevention strategies is key. Such strategies can range from individual (‘micro’) -level prevention methods, to broader national suicide prevention policies.

**Objectives:**

Whilst a range of studies have explored individual-level risk factors, highlighting municipal, regional, or national-level predictors can be valuable to identify broader social and contextual determinants. This study will therefore aim to go beyond proximal predictors of suicide by looking through a wider national- and regional-level lens in Sweden.

**Methods:**

This project will be conducted utilizing routinely collected and publicly available data and applying longitudinal modelling to investigate potential predictors of changes in suicide rates across time in Sweden. More specifically, the study will explore whether regional data on economic (e.g. proportion of state benefit recipients), socio-demographic (e.g. educational level) and healthcare related variables (e.g. trust in the healthcare system) are associated with suicide rates over time.

**Results:**

This is an ongoing project and results will be available and presented at the time of the conference.

**Conclusions:**

Utilizing publicly available data to explore potential predictors of suicide rates is not only cost-effective, but adding such findings to existing knowledge of individual-level risk factors can also be important when targeting wider policy and ensuring effective coordination and implementation of regional suicide prevention strategies.

**Disclosure of Interest:**

None Declared

